# Biofuel Cells Based on Oxidoreductases and Electroactive Nanomaterials: Development and Characterization

**DOI:** 10.3390/bios15040249

**Published:** 2025-04-14

**Authors:** Olha Demkiv, Nataliya Stasyuk, Galina Gayda, Oksana Zakalska, Mykhailo Gonchar, Marina Nisnevitch

**Affiliations:** 1 Department of Analytical Biotechnology, Institute of Cell Biology National Academy of Sciences of Ukraine, 14/16 Drahomanov Str., 79005 Lviv, Ukraine; 2 Department of Chemical Engineering, Ariel University, Kyriat-ha-Mada, Ariel 4070000, Israel

**Keywords:** biofuel cells, flavocytochrome *b*_2_, alcohol oxidase, nanomediators, power density

## Abstract

Amperometric biosensors (ABSs) and enzymatic biofuel cells (BFCs) share several fundamental principles in their functionality, despite serving different primary purposes. Both devices rely on biorecognition, redox reactions, electron transfer (ET), and advanced electrode materials, including innovative nanomaterials (NMs). ABSs and BFCs, utilizing microbial oxidoreductases in combination with electroactive NMs, are both efficient and cost-effective. In the current study, several laboratory prototypes of BFCs have been developed with bioanodes based on yeast flavocytochrome *b*_2_ (Fc*b*_2_) and alcohol oxidase (AO), and a cathode based on fungal laccase. For the first time, BFCs have been developed featuring anodes based on Fc*b*_2_ co-immobilized with redox NMs on a glassy carbon electrode (GCE), and cathode-utilizing laccase combined with gold–cerium–platinum nanoparticles (nAuCePt). The most effective lactate BFC, which contains gold–hexacyanoferrate (AuHCF), exhibited a specific power density of 1.8 µW/cm^2^. A series of BFCs were developed with an AO-containing anode and a laccase/nAuCePt/GCE cathode. The optimal configuration featured a bioanode architecture of AO/nCoPtCu/GCE, achieving a specific power density of 3.2 µW/cm^2^. The constructed BFCs were tested using lactate-containing food product samples as fuels.

## 1. Introduction

With the rising global demand for energy, interest in innovative power generation methods is increasing. Among the most promising alternatives for sustainable energy production are biofuel cells (BFCs). BFCs use enzymes and living organisms instead of conventional catalysts and offer advantages such as selectivity, specificity, and biocompatibility [1,2,3,4]. Advances in medical sciences have also led to a rising need for implantable devices such as pacemakers, insulin pumps, sensors, and prosthetic parts. These devices require reliable renewable energy sources, ideally ones that generate power from substances found in biological fluids [5,6,7,8].

Enzymatic BFCs utilize biochemical reactions of enzymes to transform chemical energy into electricity. In these BFCs, a fuel (substrate) undergoes oxidation catalyzed by an enzyme at the anode, releasing electrons that travel through an external circuit to the biocathode. On biocathodes, commonly, an oxidant like O_2_ is reduced, generating an electric current based on the potential difference, resulting in the production of electric power. The power output depends on both biological and electrochemical processes [1,9,10,11]. The first electrochemical self-powered sensing system was introduced in 2001 by Katz et al. [5].

The nature of employed catalysts in enzymatic BFCs allows the utilization of numerous fuels including a variety of sugars, organic acids, low aliphatic alcohols, and others, due to their high abundance in nature and availability at low prices on the market [12,13,14,15,16,17,18,19,20,21,22,23,24]. Lactate is of particular interest as a fuel for BFCs since biotechnological methods for its synthesis are available, and industrial waste containing this compound is accessible. Moreover, lactate is naturally present in bodily fluids such as blood and sweat [12,13,14,15,16]. Primary alcohols are also reported as viable fuels in BFCs [17,18,19,20,21]. However, the most studied and practically used are BFCs with glucose as a fuel. A search for “glucose biofuel cells” on Google Academia yields approximately 187,000 results, on PubMed—897 publications, reflecting the extensive research and interest in this field [25,26,27,28,29].

Enzymatic BFCs and amperometric biosensors (ABSs) share several fundamental principles in their functionality, despite serving different primary purposes. Biosensors are important analytical tools for healthcare monitoring, environmental monitoring, food safety, and biodefense [30]. Over the years, ABSs have been powered by external sources such as batteries and supercapacitors, as well as by self-sustaining energy sources like triboelectric, piezoelectric, and BFCs. Both systems rely on biochemical reactions, typically enzyme-catalyzed, to convert biological interactions into measurable electrical signals. Despite their distinct objectives, ABSs for analyte detection and BFCs for energy conversion, both systems rely on biorecognition, redox reactions, electron transfer, and advanced electrode materials, including innovative nanomaterials (NMs), carbon nanotubes, graphene, and metal nanoparticles. The integration of BFCs into ABSs is a promising area of research, potentially leading to self-powered, continuous monitoring devices for healthcare and environmental applications. Combining natural enzymes, especially oxidases, with suitable electroactive mimics of peroxidase (PO) offers a promising strategy for developing cost-effective enzymatic BFCs with improved characteristics, such as high stability and increased power density. The utilization of NMs for enhanced sensing performance plays a crucial role in the field of BFCs and electrochemical sensors [1,6,26,29,30,31].

BFCs have the same principles as the ABSs capable of detecting target analytes, including lactate and primary alcohols. Enzymatic detection relies on oxidoreductases to generate electrical signals proportional to analyte levels. Non-enzymatic methods typically use NMs or conductive polymers for direct oxidation of target substances. These self-powered ABSs convert biochemical energy into electrical signals, allowing simultaneous analyte detection and power generation [1,2,3,4].

Over the past 25 years, since the first concept of the self-powered electrochemical sensor was proposed in 2001 [12], BFCs have undergone significant advancements. In the past decade, there has been a technological breakthrough in the field of wearable devices [9,10,11,30]. Despite these advantages, BFCs still encounter significant challenges, such as low power densities, limited long-term stability, and biofouling in implant applications. To overcome these obstacles, extensive research efforts have focused on enhancing biocatalysts and developing advanced fabrication techniques [1,2,3,4]. Further research is needed to overcome certain limitations and establish self-powered ABSs as essential tools for the future.

The main idea of our research is the creation of new biocatalysts based on oxidoreductases and NMs for the construction of enzymatic BFCs. We plan to exploit various electro- and catalytically active NMs, including nanoparticles (NPs) of metals, as carriers for enzyme immobilization and mediators of electron transfer (ET). We have recently demonstrated that the substitution of natural enzymes, specifically, PO and laccase, with their mimics—NPs of noble and transition metals—resulted in significant amplification of the electrochemical signal, a higher stability of mediator-less ABSs, as well as a wider linearity range of analyte detection and a higher sensitivity of the bioelectrode [31,32,33,34,35,36,37].

According to our preliminary results, some compositions of two- and three-component-hybrid NPs would be promising chemo-sensors on hydrogen peroxide [32,34]. NPs were synthesized via different methods and characterized in the aspect of their catalytic parameters, selectivity of responses to the relevant substrates, and functional and storage stability [32,33,34,35,36,37,38]. Optimized bioselective membranes, containing oxidoreductases, immobilized on the most effective NPs, have been tested as sensor elements of ABSs for the assay of practically important analytes—glucose, L-lactate, and primary alcohols. The listed chemicals are important metabolites in clinical diagnostics and key components in food products. The same analytes (as fuels) and correspondent enzymes (as biocatalysts), bound with different electro- and catalytically active metallic NPs, may be promising for the development of BFC.

The implementation of enzymes into BFCs allows the development of membrane-less and compartment-less devices, which can be easily miniaturized and be useful in situations where it is not feasible to separate the fuel and oxidant. For these applications, the achievement of efficient ET between the enzyme active center and the electrode is critical. Usually, for mediated ET there are used small redox active NPs and polymers as electron carriers (mediators), for example, organic dyes (methylene blue, etc.), metalorganic compounds (ferrocene, etc.), co-enzymes and conducting compounds—salts or polymers (polyaniline, etc.). If the mediators are present in solution as free-diffusing mediators (FDM), their movement toward the bioelectrode surface facilitates ET more effectively than direct ET from the enzyme itself [33].

Enzymatic biocathodes hold considerable potential to replace platinum as an expensive catalyst in fuel cells (FCs). Among the most promising enzymes for biocathodes is laccase, which can oxidize a broad spectrum of substrates, such as phenols and other aromatic compounds commonly found in fuel sources, including chemical industry waste. However, these biocathodes face challenges such as slow ET rates from the enzyme, low cathode stability, and a limited operational lifespan.

These shortcomings can be addressed by optimizing the immobilization process, which enhances direct ET, increases enzyme activity, and improves its stability. Various approaches for developing biocathodes with improved functional properties have been described in the literature, utilizing auxiliary materials such as nanotubes, osmium-based redox polymers, polylysine, or graphite [1,2,3,4,5]. It is well known that the use of mediators significantly improves ET between the electrode surface and the enzyme.

To develop a novel BFC, we plan to use fungal laccase, yeast alcohol oxidase (AO), flavocytochrome *b*_2_ (Fc*b*_2_), and electroactive NMs as mediators of ET. All these enzymes have been used earlier as bioelements of ABSs [32,33,34,35,36,37,38]. Special attention was paid to studying the possibility of direct (mediator-less) electrochemical communication between enzyme and electrode surface in redox systems.

## 2. Materials and Methods

### 2.1. Reagents and Enzymes

2,2′-azino-bis (3-ethylbenzothiazoline-6-sulfonic acid (ABTS), cetyltrimethylammonium bromide (CTAB), Nafion, primary alcohols, hydrogen peroxide, inorganic salts, polyvinylpyrrolidone (PVP) and all other chemicals of analytical grade were supplied by Sigma-Aldrich (Steinheim, Germany). All buffer and substrate solutions were prepared in ultrapure water from the Milli-Q^®^ IQ 7000 Water System, produced by Merck KGaA (Darmstadt, Germany).

Enzymes were purified and characterized according to the methods developed by the authors [37,39]. Highly purified flavocytochrome *b*_2_ (Fc*b*_2_, EC 1.1.2.3) and alcohol oxidase (AO, EC 1.1.3.13) were isolated from the yeast *Ogataea polymorpha* [39]. Laccase was isolated from the fungus *Trametes zonata* [37]. Enzyme activities were measured using a Shimadzu UV-1650 PC spectrophotometer (Kyoto, Japan).

### 2.2. Synthesis of Metallic Nanoparticles

The synthetic procedures for several NPs have been detailed in our previous publications: nCuPd [32], hexacyanoferrates of gold (AuHCF) [33] and copper (nCuHCF) [34], nCoCuCe [35], nAuPt [36], nCuPt [40], nAuCePt [41], and nCu [40,42].

The synthesis of novel NPs, including nCo, nCoPtCu, nPtPd, and nAgCePt, is reported in this manuscript.

The synthesis of nCoPtCu involves three stages of a sequential reduction process, utilizing ascorbic acid as a reducing agent. nCo were first obtained by chemical reduction using ascorbic acid as a reducing agent. Initially, 0.5 mL of 0.05 M CoSO_4_ was added to 10 mL of a 1 mM CTAB solution and stirred for 5 min. Subsequently, 1 mL of 0.05 M ascorbic acid was introduced into the mixture, which was then vigorously stirred and heated at 100 °C for 10 min. The resulting nCo were collected by centrifugation (7000× *g*, 30 min), washed with 5 mM phosphate buffer (pH 7.0), and rinsed with water. The synthesized yellow nCo was used as seeds for the second stage.

5 mL of the nCo solution was mixed with 1 mL of 0.02 M H_2_PtCl_6_ and 5 mL of 0.01 M ascorbic acid. The mixture was heated at 100 °C for 10 min. The resulting yellow nCoPt, after concentration and washing, was used as seeds for the third stage.

5 mL of the nCoPt solution was mixed with 10 mL of 0.05 M CuSO_4_ and 5 mL of 0.02 M ascorbic acid. The mixture was heated at 100 °C for 10 min, and the resulting nCoPtCu precipitate was collected by centrifugation (7000× *g*, 30 min), washed with 5 mM phosphate buffer (pH 7.0), water, and dried at 100 °C for 24 h.

The synthesis of nAgCu was carried out as follows: equal volumes of 5 mM CuSO_4_ and AgNO_3_ solutions were mixed in a 1:1 ratio. Sodium citrate was then added to the mixture to achieve a final concentration of 0.1 mM. The resulting solution was stirred vigorously at room temperature for 30 min. Following this, sodium borohydride was added dropwise as a reducing agent, causing a visible color change that signaled the formation of nanoparticles. The reaction mixture was then stirred for an additional 2 h while being heated to 50 °C.

The synthesis of nPtPd was provided using the borohydride reduction method. A 10 mL solution of 1 mM H_2_PtCl_6_·H_2_O and 10 mL of 1 mM PdCl_2_ were mixed, followed by the addition of 0.1 mL of 10% PVP under vigorous stirring for 1 h. Subsequently, a 2.5 mM NaBH_4_ solution was added as a reducing agent. The final product was washed multiple times with ethanol and deionized water to remove impurities.

The synthesis of nAgCePt involves a sequential reduction process, utilizing NaBH_4_ as a reducing agent. Initially, 2 mL of 2% AgNO_3_ solution was mixed with 2 mL of 2% CeCl_3_ solution, followed by vigorous stirring for 20 min at 20 °C. Subsequently, 0.02 mL of 10 mM NaOH was added, and the resulting mixture was incubated under stirring for 2 h at 20 °C. The formed nAgCe were collected by centrifugation, washed with water, and suspended in 2 mL of water. Independently, 2 mL of 2% H_2_PtCl_6_ was vigorously stirred with 0.1% CTAB for 15 min at 20 °C, mixed with 2 mL of nAgCe and 0.1 mL of 100 mM NaBH_4_. The reaction mixture was incubated under stirring for 24 h at 20 °C.

The obtained NPs were concentrated by centrifugation, the precipitates were washed twice with water and stored as a water–colloid solution at +4 °C or in form dried until use.

### 2.3. Characterization of Metallic Nanoparticles

The structure and morphology of NPs were examined using a REMMA-102-02 SEM microanalyzer (Selmi, Sumy, Ukraine) [32,33].

To select catalytically active nanoparticles (NPs), we screened the synthesized NPs for their laccase-like and peroxidase-like (PO-like) activities. For this purpose, we employed a naked-eye colorimetric semi-quantitative approach using 1 mM ABTS as a chromogenic substrate [38]. Each testing tube contains 5 µg of the NP sample in 1 mL of substrate solution.

The assay of PO-like activity was performed in the presence of 0.1 mM H_2_O_2_ in ABTS. The quantitative determination of both catalytic activities has been described in our recent work [32]. One unit (U) of PO-like activity is defined as the amount of NZ that releases 1 µmol of H_2_O_2_ per minute under standard assay conditions.

To select the optimal electroactive mediator, an aliquot of the synthesized NPs was immobilized on the GCE surface. The dried NP layer was coated with a 1% Nafion solution, then dried and rinsed with buffer. The redox activity of the constructed NPs/GCE was studied using cyclic voltammetry (CV) in a 10 mM K_3_[Fe(CN)_6_] solution containing 0.1 M KCl.

### 2.4. Apparatus and Measurements

To develop two-compartment enzyme fuel cells (BFCs) as lactate and ethanol-powered sensors, glassy carbon electrodes (GCE) were used. Amperometric experiments were conducted as described in our previous papers [32,33,37], using a Pt wire as the counter electrode, an Ag/AgCl/3M KCl electrode as the reference, and a GCE with a diameter of 3.0 mm as the working electrode. The development and characterization of amperometric (bio)electrodes, along with the statistical analysis of measurements, have been performed as previously described [32,33].

All experiments were performed in triplicate, with measurements in two parallels. Statistical parameters and figures were plotted and generated using Origin 8.5 Pro.

### 2.5. Fabrication of the Enzymatic Fuel Cell

A bioelectrode consisted of oxidoreductases (enzyme/GCE), both unmodified (control) and modified with various electroactive NPs (enzyme/NPs/GCE). To prepare bioanode, solutions of AO (5 µL, 50 U/mL) or Fc*b*_2_ (5 µL, 10 U/mL) in 50 mM phosphate buffer, pH 7.5, were immobilized on the surface of GCE or NPs/GCE. To obtain a biocathode, a laccase in sulfate ammonium suspension (2 µL, 20 U/mL) in 50 mM acetate buffer, pH 4.5 was deposited on the GCE or NPs/GCE as described earlier [42]. The obtained bioelement on the surface of GCE was covered with 1% Nafion.

The constructed BFC was based on two previously modified GCEs with a surface area of 7.07 mm^2^, which were immersed into two compartments containing solutions of different compositions. The AO-based and Fc*b*_2_-based bioanodes were immersed in 10 mL 50 mM phosphate buffer pH 7.5 containing different concentrations of lactate and ethanol, respectively.

The laccase-based biocathode was immersed in 10 mL 50 mM acetic buffer containing 1 mM ABTS. The anode and cathode cells were linked through a salt bridge containing a saturated solution of KCl. The characteristics of the constructed BFCs, including current density, power density, and open-circuit voltage (OCV), were determined as described earlier [42]. All prepared bioelectrodes were rinsed by correspondent buffers and kept at +4 °C before usage.

## 3. Results and Discussion

### 3.1. General Principle of FBC Functionality

To develop a novel BFC, we used fungal laccase, yeast AO, and Fc*b*_2_. These enzymes have been used earlier as bioelements of ABSs [32,33,34,37,38,39].

Laccase is one of the most promising enzymes for biocathode. Laccase oxidizes a broad spectrum of substrates, such as phenols and other aromatic compounds commonly found in fuel sources, including chemical industry waste [2,3,43,44].

Bioanodes that use ethanol or other low-molecular-weight primary alcohols as fuel are typically composed of alcohol dehydrogenase in the presence of its co-factor NAD^+^ [18,21,43,45,46,47,48,49]. Another type of BFCs is based on AO [21,44,46,50,51].

Lactate-utilizing bioanodes are typically composed of lactate oxidase or lactate dehydrogenase in the presence of its cofactors NAD(P) or FMN [12,13,14,15,16]. To date, there are no reports on the use of Fc*b*_2_ in the fabrication of BFCs.

To enhance the performance of BFCs, electroactive materials with high surface area and catalytic activity are commonly employed. Additionally, oxidase-based bioanodes often contain natural or artificial PO co-immobilized with oxidase [50].

**Figure 1:**
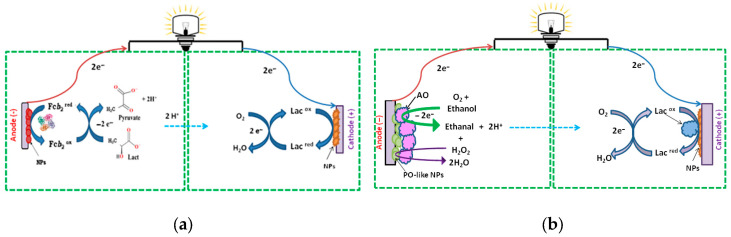
Schematic representation of the mechanisms of BFC function with Fc*b*_2_ (**a**) and AO (**b**) at the anodes, and laccase (Lac) at the cathodes (**a**,**b**).

Figure 1 illustrates the mechanism of BFC operation and general reactions occurring at both bioelectrodes in enzymatic BFCs.

### 3.2. Construction and Optimization of Laccase-Based Biocathodes

To develop an effective laccase-based cathode, the selected NPs with high redox activity were explored. A series of noble and transition metals MPs were synthesized using various methods. After immobilization on the surface of GCEs, the resulting NPs/GCEs have been screened for their electroactivity using cyclic voltammetry (CV). All tested NPs were demonstrated to have electro-mediator activity, as the oxidation and reduction peaks of the tested NPs/GCEs were higher compared to the unmodified control electrode.

**Figure 2:**
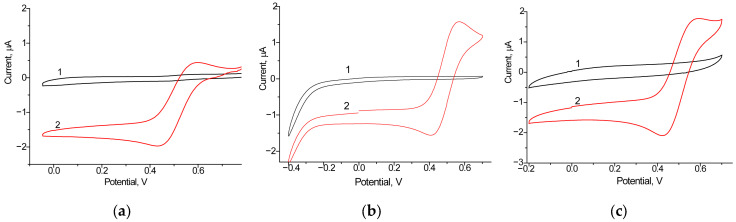
Cyclic voltammograms of constructed laccase-based biocathodes, unmodified (**a**) and modified with nanomediators nAuCePt (**b**) and nCuPt (**c**) without the addition (1, black line) and after the addition of 1 mM ABTS (2, red line). Conditions: scan rate of 20 mV·s^−1^ vs. Ag/AgCl/3M KCl, 50 mM acetate buffer, pH 4.5.

We screened eight bioelectrodes with the architecture laccase/NPs/GCE, using the following NPs: nAuCePt, nCu, nCo, nCuS, nCoPt, nCuPt, nPtCe, and CuHCF. Among the tested bioelectrodes, the most significant increase in both oxidation and reduction currents was observed for two variants (laccase/nCuPt/GCE and laccase/nAuCePt/GCE), indicating enhanced electron communication at the electrode surface. The CV profiles of the most effective electrodes are presented in Figure 2, while Figure S1 demonstrates less significant results. As shown in these figures, the highest oxidation and reduction peaks were observed for the single biocathode, laccase/nAuCePt/GCE, in the presence of 1 mM ABTS, a substrate of laccase. It is important to note that the ET process between biomolecules and the electrode surface is a key factor in achieving high performance in BFCs. Therefore, laccase/nAuCePt/GCE was selected for further use as the biocathode in the development of various BFCs.

To select NPs with the highest catalytic activity, namely, laccase-like and PO-like, a colorimetric naked-eye screening test was used [38]. Results are presented in Table S1 and Figure S2. As shown in Figure S2, nAuCePt, nCuPt, and nCoPt exhibit the most effective laccase-like properties in solution. Therefore, these NPs may be promising for enhancing the catalytic capability of natural laccase when co-immobilized on the surface of the biocathode.

### 3.3. Development and Optimization of AO-Based Bioanode

Alcohol oxidase (AO) in combination with metallic NPs was used to design the bioanode. The redox behavior of the designed bioelectrodes was investigated by the CV.

**Figure 3:**
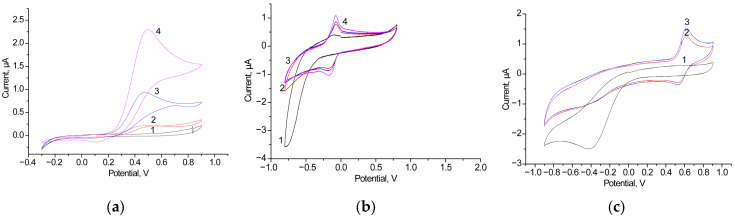
CV profiles as outputs on the added ethanol up to 0 mM (black line, (**a**–**c**)), 0.5 mM (blue line, (**a**)–(**c**)), 1 mM (red line, (**a**–**c**)) and 2 mM (pink line, (**a**–**c**)) for the bioanodes AO/GCE in the presence of various 1 mM FDMs: HQ (**a**), methylene blue (**b**) and ferrocene (**c**). Conditions: scan rate of 20 mV·s^−1^ vs. Ag/AgCl/3M KCl, 50 mM phosphate buffer, pH 7.5.

Our first task was to select the most effective free-diffusing mediator (FDM) for the AO/GCE. Figure 3 presents a comparative study of the CV profiles of AO/GCEs with hydroquinone (HQ), methylene blue, and ferrocene as the FDMs upon the addition of 1 mM ethanol (EtOH) as a substrate for AO.

As shown in the results presented in Figure 3, HQ is the most effective FDM. Therefore, further experiments on the development of BFCs with AO-based anode were conducted using HQ.

**Figure 4:**
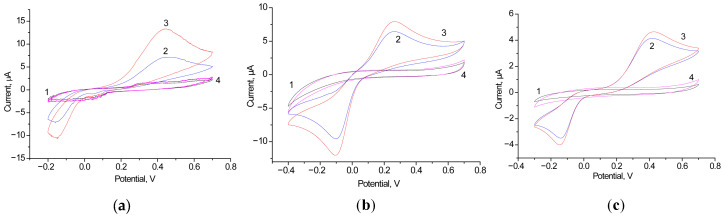
CV profiles in the presence of 1 mM HQ were measured for various bioelectrodes with the architecture AO/NPs/GCE (lines 1–3) and for the control electrode without AO (line 4), with ethanol concentrations of 0 mM (1, black line), 0.5 mM (2, blue line), and 1 mM (3, red line). The compositions of the NPs were as follows: nAgCu (**a**), nPdPt (**b**), and nCoCuCe (**c**). The control electrodes, NPs/GCEs, were tested without added ethanol (pink line, 4). Measurements were conducted at a scan rate of 20 mV·s^−1^ vs. Ag/AgCl/3M KCl in 50 mM phosphate buffer, pH 7.5.

The next task was to screen different electroactive metallic NPs concerning their capability to effective ET under the AO catalysis of EtOH oxidation. We have screened several NPs as PO-like nanomediators. The redox behavior of the designed bioelectrodes was investigated by the CV. The electrochemical response of the most effective electrodes with the architecture of AO/NPs/GCE to the addition of ethanol is shown in Figure 4. It was demonstrated that the redox mediator HQ does not influence the CV profiles of the control electrodes NPs/GCE in the absence of EtOH. The described bioanodes, AO/nAgCu/GCE, AO/nPdPt/GCE, AO/nCoCuCe/GCE, and several others, were used for the construction of BFCs.

### 3.4. Construction of AO-Based BFCs

**Figure 5:**
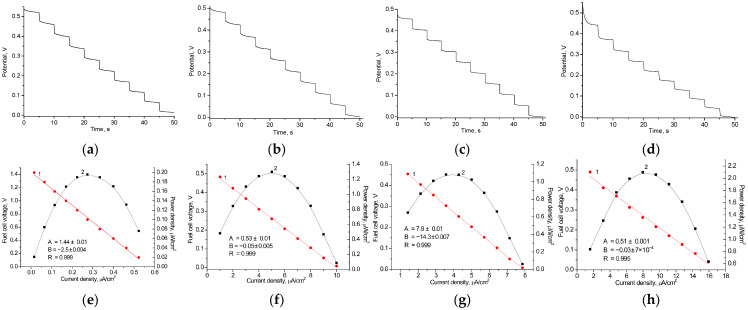
Working characteristics of various AO/NPs-based BFCs in the presence of 1 mM EtOH and HQ: graphs of time-dependent potential loss (**a**–**d**), polarization curves (1, red) and power density curves (2, black) (**e**–**h**). NPs: control, without NPs both on anode and cathode (**a**,**e**); nAgCu (**b**,**f**); nAgCePt (**c**,**g**); nCoCuCe (**d**,**h**).

AO, in combination with various synthesized metallic NPs as well as with commercial carbon nanotubes (CNTs), was used to design the bioanodes with the architecture of AO/NMs/GCE, while laccase immobilized with a nanomediator on the electrode surface (laccase/nAuCePt/GCE) was used as a biocathode. The main working characteristics of the most effective BFCs, which include open-circuit voltage (OCV), power density, and current density, are shown in Figure 5. Table 1 summarizes these results.

AO/nCoCuCe/GCE and AO/nCoPtCu/GCE demonstrate higher power densities (2.10 and 3.20 µW/cm^2^, respectively) compared to other tested AO/NPs-based bioanodes. This can be attributed to the positive effect of the NPs in the composition of the bioelectrode. Being enzyme carriers in bioanode with architecture AO/NPs/GCE, these redox-active NPs are the most effective PO mimics (see Figure S3). By decomposing H_2_O_2_, these NPs enhance the efficiency of the AO-mediated ethanol oxidation reaction. Figure S4 shows SEM images of nCoPtCu and nCoCuCe.

Thus, ethanol-dependent BFCs were proposed using enzymes coupled with electroactive nanomediators, with AO on the anode and laccase on the cathode. Although the specific power densities of the developed BFCs are relatively low compared to alcohol dehydrogenase-based BFCs, the open-circuit voltage and current are significant. It is worth noting that our study demonstrates the possibility of creating novel devices using microbial enzymes and newly developed NMs.

Many reports have been published on methods to improve the performance characteristics of BFCs and on explaining the underlying functional mechanisms [1,2,3,4,5,44,45,46,47,48,49,50,51,52]. To achieve high power output in the BFC, it is essential to improve the enzyme immobilization technology on the electrode surface to enhance the enzyme-electrode electron transfer [1,2,3,4,5]. It is necessary to obtain novel recombinant enzymes with enhanced catalytic characteristics and to synthesize stable NMs with advanced surface area [48]; to select the optimal enzyme combinations, quantities, and the enzyme/NM ratio on the electrode surface [10,11]; to use small-size cells [49]; to study the dependence of BFC power output on fuel concentration [18,44,47]; and the optimal operating conditions for the BFC, including pH, salt composition, temperature, viscosity and oxygen concentration [18,49].

### 3.5. Creation of the Fcb_2_-Based BFC

The aim of this part of the study was to develop a BFC for energy generation using L-lactate (hereafter referred to as lactate) as a fuel. In the BFC, Fc*b*_2_ was immobilized on the anode to oxidize lactate, while laccase was immobilized on the cathode to catalyze the reduction of molecular oxygen to water, accompanied by the oxidation of the substrate ABTS. A schematic representation of the developed BFC configuration is provided in Figure 1a. The most effective bioelectrode, laccase/nAuCePt/GCE, was selected as the cathode (see Section 3.1).

For BFC fabrication, we selected electroactive mediators to enhance ET between Fc*b*_2_ and the electrode surface. It is known that Fc*b*_2_ is capable of direct ET; however, without additional mediators, the resulting electric signal is very weak [33,36].

**Figure 6:**
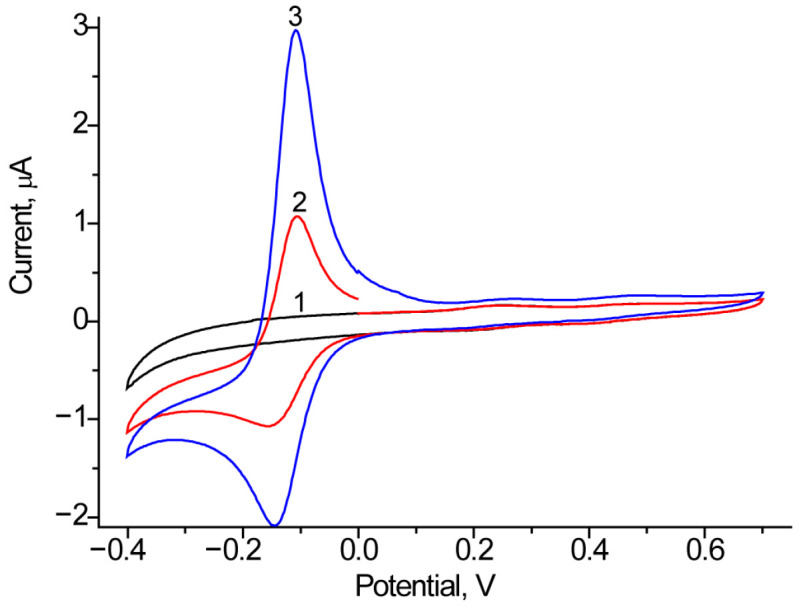
CV profiles as outputs on the added lactate up to 0 mM (1, black line), 1 mM (2, red line) and 2 mM (3, blue line) for the bioanode Fc*b*_2_/GCE in the presence of 1 mM PMS. Conditions: scan rate of 20 mV·s^−1^ vs. Ag/AgCl/3M KCl, 50 mM phosphate buffer, pH 7.5.

Using the CV approach, we screened FDMs, which were introduced into the electrode bath solution. As FDMs, we tested 1 mM PMS, HQ, and K_3_Fe(CN)_6_, with PMS and HQ demonstrating the best performance. As an example of the influence of PMS on ET, Figure 6 presents the CV profiles of Fc*b*_2_/GCE both with and without PMS.

**Figure 7:**
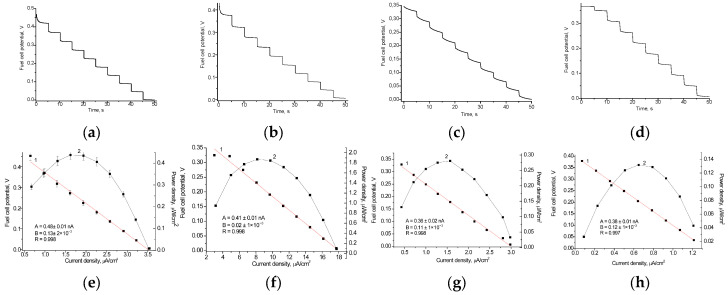
Working characteristics of various Fc*b*_2_/NPs-based BFCs in the presence of 1 mM lactate: graphs of time-dependent potential loss (**a**–**d**); polarization curves (1, red), and power density curves (2, black) (**e**–**h**). Mediators: nAuHCF (**a**,**e**); nAuHCF with HQ (**b**,**f**); nAuPt with PMS (**c**,**g**); nAuPt with K_3_Fe(CN)_6_ (**d**,**h**).

We tested several electroactive metallic NPs as nanomediators in a Fc*b*_2_-based BFCs. In our previous studies, we demonstrated that nAuHCF [33] and nAuPt [36] are the most effective redox mediators for the development of Fc*b*_2_-based ABSs. Thus, in the current study, we utilized these NPs to construct a lactate-dependent bioanode. These results are presented in Figure 7 and summarized in Table 2.

According to the results presented in Table 2, the OCV values of the constructed BFCs range from 0.35 V to 0.54 V, depending on the type of anode, while the fuel cell’s operating potential ranges from 0.35 V to 0.44 V. The most effective lactate BFC consists of Fc*b*_2_/nAuHCF/GCE with HQ as the mediator on the anode and laccase/nAuCePt with ABTS on the cathode. This BFC exhibits the highest power density (1.8 μW/cm^2^) and short-circuit current (550 nA).

Thus, lactate BFCs were developed for the first time using enzymes coupled with electroactive nanomediators, with Fc*b*_2_ on the anode and laccase on the cathode. Although the specific power density of the developed BFC is rather low compared with lactate oxidase- and lactate dehydrogenase based BFCs, the open-circuit voltage and open-circuit current are significant. In the future, attention should be focused on improving the immobilization technology of enzymes on the anode surface to enhance enzyme-electrode communication, which plays a crucial role in achieving high power output in the BFC.

### 3.6. Testing the Developed Fcb_2_-Based BFC on Real Food Products as Lactate-Containing Fuel

To evaluate the power output of the fabricated BFCs, real food products that contain lactate were used. For this aim, the constructed Fc*b*_2_/nAuPt/GCE bioanode was immersed in the tested samples (in particular, yogurt or cucumber brine), and the main electrochemical parameters of BFCs were studied. In our previous paper [36], we determined the lactate contents in these products, using Fc*b*_2_-based ABSs; these values were 74 mM in yogurt and 93 mM in cucumber brain.

**Figure 8:**
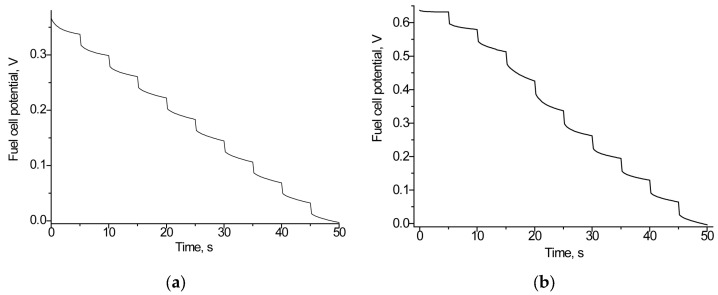

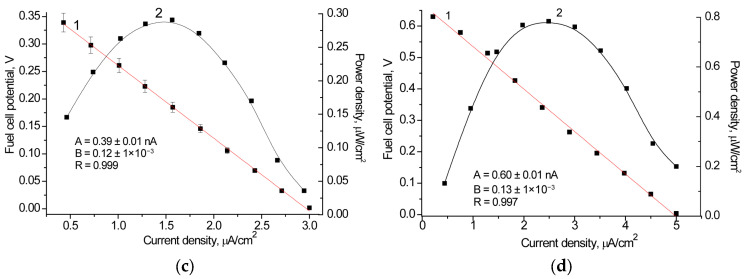
Working characteristics of the BFC generating current from lactate-containing yogurt (**a**,**c**) and cucumber brine (**b**,**d**): time-dependent potential loss graphs (**a**,**b**); polarization curves (1, red), and power density curves (2, black) (**c**,**d**). The BFC consisted of an Fc*b*_2_/nAuPt/GCE anode with PMS as the mediator and a laccase/nAuCePt/GCE cathode with ABTS as the mediator.

Figure 8 demonstrates the capability of the studied BFC to generate electricity when using food products as lactate sources. Table 3 summarizes the results obtained.

## 4. Conclusions

Enzymatic biofuel cells were created using purified oxidoreductases and novel nanomaterials, with their functional parameters thoroughly investigated. NMs were synthesized via the sol–gel method and characterized for conductivity and catalytic activity, especially, pseudo-peroxidase and pseudo-laccase. To develop a BFC with enhanced performance and stability, enzymes were co-immobilized with the most promising electroactive NMs, which have a high surface area and act as ET mediators on electrode surfaces: yeast oxidoreductases AO and Fc*b*_2_ on the anodes, and fungal laccase on the cathode. The functional characteristics of the laboratory prototypes of the enzymatic BFCs were subsequently studied. Special attention was paid to studying the possibility of direct (mediator-less) electrochemical communication between enzyme and electrode surface in redox systems.

As a result of this investigation, two Fc*b*_2_-based BFCs were developed for the first time. Both configurations—BFC-1 and BFC-2—utilized a cathode containing laccase and nAuCePt, while their respective anodes were modified with nAuHCF and nAuPt. BFC-1 exhibited a specific power density of 1.8 μW/cm^2^, which is significantly higher than that of the Fc*b*_2_-based anode without mediators. BFC-2 was shown to be capable of generating electrical energy—albeit at a very low level—from lactate-containing food products.

Laboratory prototypes of BFCs were created with an AO-based bioanode and a laccase-based cathode, utilizing various NMs. These NMs serve as carriers for enzyme immobilization, enhancing ET efficiency and improving the BFC’s stability. The best performance was achieved with a bioanode featuring the AO/nCoPtCu/GCE architecture, which demonstrated an open-circuit potential of 0.60 V and a specific power density of 3.2 μW/cm^2^. These advancements highlight the potential of integrating specific enzyme/NM combinations to enhance the efficiency of BFCs.

## Figures and Tables

**Table 1:** Working characteristics of the most effective AO-based BFC.

Bioanode	Biocathode	OCV, V	Power Density, µW/cm^2^	Resistance, Ohm	A Current of a Short Circuit, nA
AO + ^1^ HQ	Laccase + ABTS	0.58 ± 0.02	0.20 ± 0.01	0.50 ± 0.02	100 ± 7.0
AO/^2^ CNTs + HQ		0.56 ± 0.02	0.49 ± 0.03	0.15 ± 0.01	235 ± 10
AO/nAgCu + HQ	Laccase/nAuCePt + ABTS	0.54 ± 0.01	1.30 ± 0.02	0.05 ± 0.03	700 ± 5.5
AO/nAgCePt + HQ		0.50 ± 0.03	1.04 ±0.07	0.20 ± 0.01	600 ± 3.3
AO/nPtPd + HQ		0.50 ± 0.04	1.08 ± 0.04	0.20 ± 0.01	550 ± 7.0
AO/nCoCuCe + HQ		0.57 ± 0.03	2.10 ± 0.12	0.03 ± 0.002	400 ± 2.5
AO/nCoPtCu + HQ		0.63 ± 0.05	3.20 ±0.15	0.02 ± 0.001	600 ± 5.9

^1^ HQ—hydroquinone; ^2^ CNTs—carbon nanotubes.

**Table 2:** Working characteristics of various lactate BFCs.

Bioanode	FDM	Biocathode	OCV, V	Fuel Cell Potential, V	Power Density, μW/cm^2^	Current of Short Circuit, nA
Fc*b*_2_/nAuHCF	-	laccase/nAuCePt with ABTS	0.54 ± 0.04	0.44 ± 0.03	0.44 ± 0.04	110 ± 10
	HQ		0.44 ± 0.03	0.35 ± 0.02	1.80 ± 0.1	550 ± 45
Fc*b*_2_/nAuPt	PMS		0.35 ± 0.02	0.35 ± 0.03	0.28 ± 0.02	210 ± 18
	K_3_Fe(CN)_6_		0.4 ± 0.03	0.37 ± 0.03	0.13 ± 0.01	85 ± 7

**Table 3:** Working characteristics of the lactate BFC generating current from food products.

Bioanode	Fuel	Biocathode	OCV, mV	Fuel Cell Potential, V	Power Density, μW/cm^2^	A Current of the Short Circuit, nA
Fc*b*_2_/nAuPt + PMS	yogurt	laccase nAuCePt/ABTS	0.39 ± 0.03	0.37 ± 0.03	0.30 ± 0.03	210 ± 18
	cucumber brine		0.65 ± 0.05	0.60 ± 0.04	0.78 ± 0.05	350 ± 20

## Data Availability

Data is contained within the article or Supplementary Materials.

## Supplementary Materials

The following information is available online at https://www.mdpi.com/article/10.3390/bios15040249/s1, Figure S1: CV profiles of the biocathodes with the architecture laccase/NPs/GCE without ABTS and in the presence of 1 mM ABTS; Figure S2: Visualization of the catalytic activity of NPs using ABTS. Figure S3: Visualization of the PO-like activity of NMs, including NPs and CNTs, used in AO-based bioanode construction; Figure S4: SEM images of novel NPs used in the construction of the AO-based bioanode. Table S1: Pseudo-laccase activity of nanomediators used as laccase carriers in biocathode.

## Abbreviations

ABS	Amperometric biosensor
ABTS	2,2′-azinobis (3-ethylbenzothiazoline-6-sulfonate) diammonium salt
AO	Alcohol oxidase
BFC	Biofuel cell
CTAB	Cetyltrimethylammonium bromide
Fc*b*_2_	Flavocytochrome *b*_2_
GCE	Glassy carbon electrode
HCF	Hexacyanoferrate of metal
HQ	Hydroquinone
NM	Nanomaterial
NPs	Nanoparticles
OCV	Open-circuit voltage
PMS	Phenazyne methosulfate
PO	Peroxidase
PVP	Polyvinylpyrrolidone
SEM	Scanning electron microscopy
XRM	X-ray spectral microanalysis
