# Supplementary material for: Biofuel Cells Based on Oxidoreductases and Electroactive Nanomaterials: Development and Characterization
Source: Biosensors (Basel). 2025 Apr 14;15(4):249. doi: 10.3390/bios15040249 (PMC12024903; doi:10.3390/bios15040249)
Supplement: Supplementary file 1 [file biosensors-15-00249-s001.zip › biosensors-3541067-supplementary.pdf]

Supplementary Information

Article

Biofuel Cells Based on Oxidoreductases and Electroactive Nanomaterials: Development and Characterization

Olha Demkiv [1], Nataliya Stasyuk [1], Galina Gayda [1,*], Oksana Zakalska [1], Mykhailo Gonchar [1] and Marina Nisnevitch [2,*]

[1] Department of Analytical Biotechnology, Institute of Cell Biology National Academy of Sciences of Ukraine, 14/16 Drahomanov Str., 79005 Lviv, Ukraine; gonchar@nas.gov.ua (M.G.)

[2] Department of Chemical Engineering, Ariel University, Kyriat-ha-Mada, Ariel 4070000, Israel

* Correspondence: galina.gayda@nas.gov.ua or galina.gayda@gmail.com (G.G.); marinan@ariel.ac.il (M.N.)

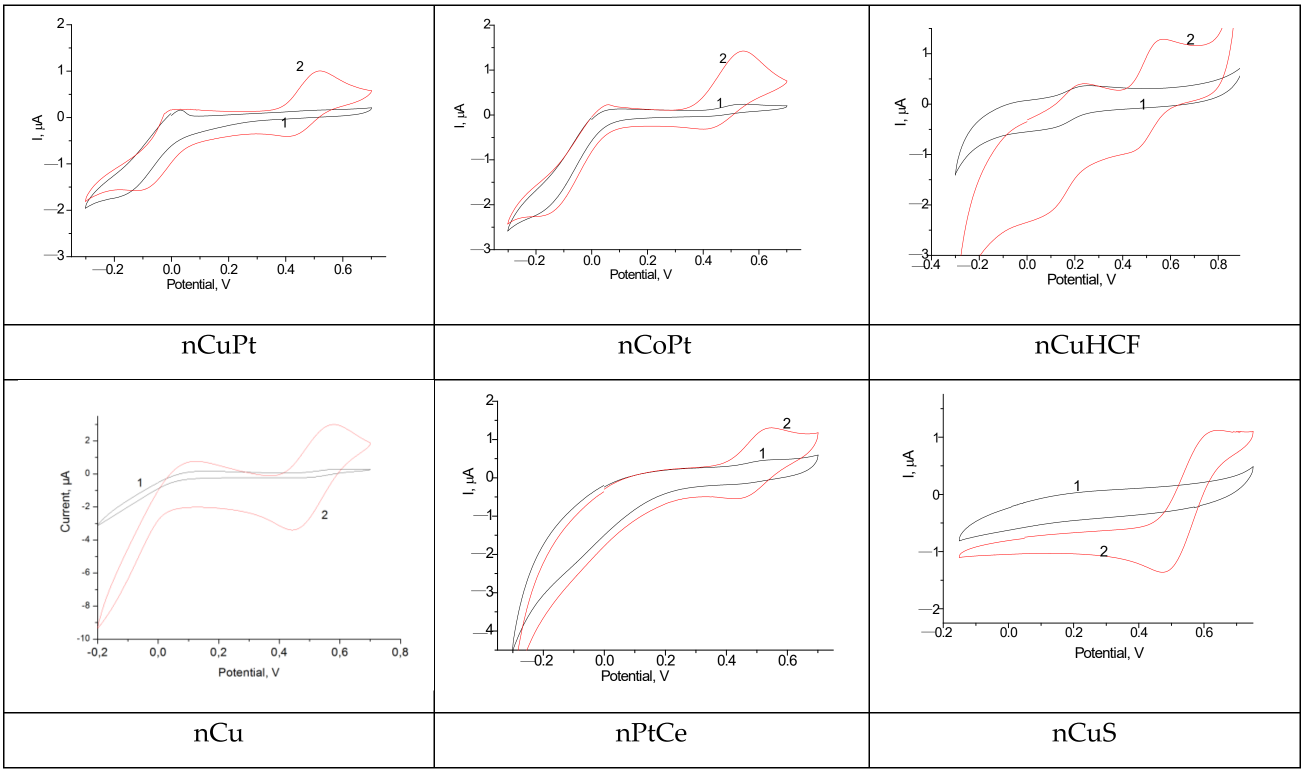

Figure S1. CV profiles of the biocathodes with the architecture laccase/NPs/GCE without ABTS (1, black) and in the presence of 1 mM ABTS (2, red).

Table S1. Pseudo-laccase activity of nanomediators used as laccase carriers in biocathode.

Number	NPs	Activity, U/mg
1	Control	0
2	nAuCePt	4.2
3	nCuPt	3.5
4	nCoPt	2.0
5	nCo	1.3
6	nCuHCF	1.3
7	nCu	0.35
8	nPtCe	0.4
9	nCuS	0.3

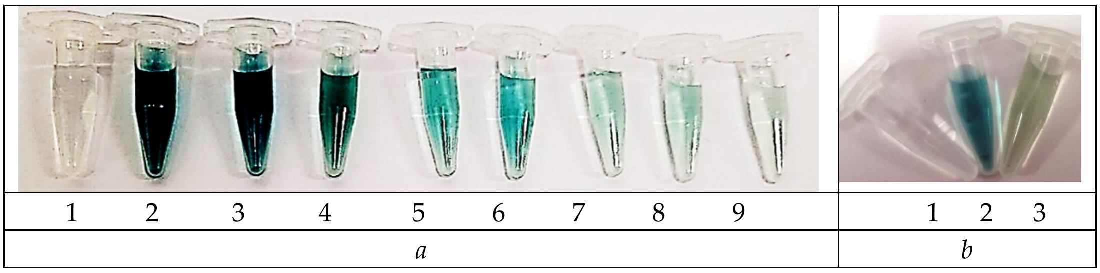

Figure S2. Visualization of the catalytic activity of NPs using ABTS. Tube 1 (a, b) serves as the control without NPs, while tubes 2-9 contain 5 μg of the correspondent NPs samples listed in Table S1. Pseudo-laccase activity is visualized for samples (2-9) in the image (a) and for sample 2 in the image (b). Tube 3 in panel (b) illustrates the pseudo-PO activity for sample 2.

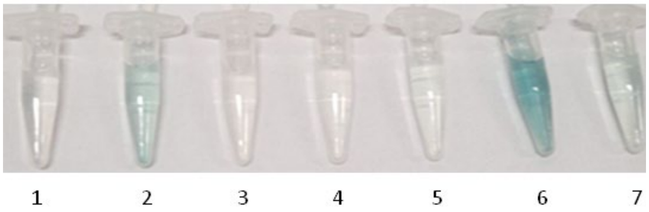

Figure S3. Visualization of the PO-like activity of NMs, including NPs and CNTs, used in AO-based bioanode construction. Each tube contains 5 μg of the NMs sample in 1 mL of substrate solution. The samples are as follows: control without NPs (1); nCoCuCe (2); CNTs (3); nPtPd (4); nAgCePt (5); nCoPtCu (6); nAgCu (7).

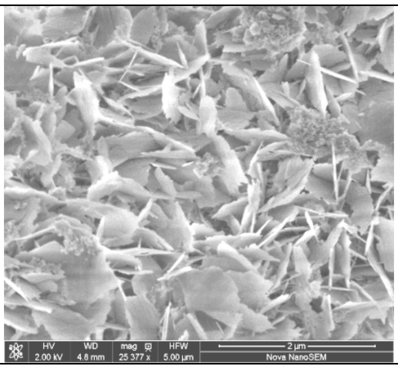	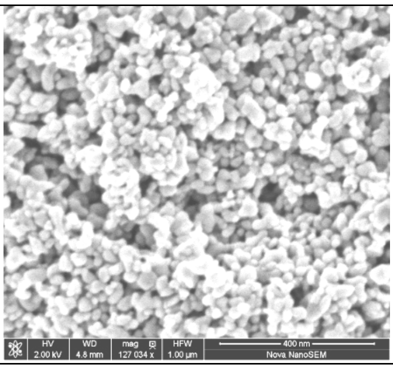
nCoPtCu	nCoCuCe
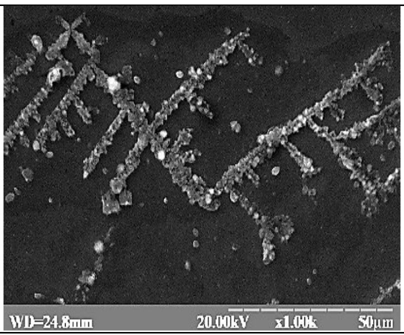	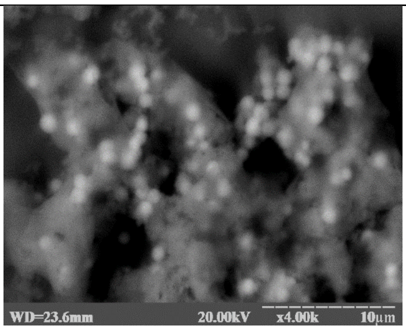
nAgCu	nPtPd

Figure S4. SEM images of the novel NPs used in the construction of the AO-based bioanode.
